# Surglcel ^®^ Reinforced Resection Lines in Left-sided Hepatectomy with Linear Stapling Device. An Experimental Study on Pigs

**DOI:** 10.1155/1996/86936

**Published:** 1996

**Authors:** Thomas Zilling, Torsten Holmin

**Affiliations:** Department of Surgery Lund University Hospital Sweden

## Abstract

Fourteen pigs underwent left-sided hepatectomy. The resection was performed with a linear stapling device and the pigs were randomised to either Surgicel ^®^ reinforced resection lines or not. The median time required for resection was 25 min (range 17-30) in the Surgicel^®^ reinforced group compared to 30 min (range 21-41) in the stapled group. This difference was, however, not statistically significant (p=0.053).

The postoperative haemoglobin value was lower in the stapled group compared to the Surgicel^®^ reinforced group 69 g/1 (range 42-85) versus 82 g/1 (range 78-90) (p=0.018). The estimated blood losses by weighing the compresses were 287 ml (range 166-379) for the stapled group and 204 ml (range 152-264) for the Surgicel^®^ reinforced group (p=0.053). The median number of additional haemostatic sutures in the Surgicel^®^ reinforced group was 7 (range 3-11) and in the stapled group 10 (range 5-15) (p=0.038).

The haemoglobin value was similar in the two groups week postoperatively; 100 g/1 (range 87-104) and 102 g/1 (range 95-114), p=0.27, in the stapled group and the Surgicel^®^ reinforced group, respectively. In the stapled group reinforced with the Surgicel^®^ there was one postoperative death. In the solely stapled group there was no postoperative death (p=0.5). Four out of six pigs in the Surgicel^®^ group had massive adhesions to the resection lines. One of these six pigs was sacrificed postoperatively as it was ill and had small bowel obstruction secondary to Surgicel^®^ induced adhesions. On the other hand, no adhesions were seen in the solely stapled pigs (p=0.09). At this point, we can not recommend the use of Surgicel^®^ to reinforce resection lines at stapled liver resection in the clinical situation, because of the high frequency of adhesions this material creates.


*HPB Surgery*, 1996, Vol. 9, pp. 97–99
Reprints available directly from the publisher
Photocopying permitted by license only

© 1996 OPA (Overseas Publishers Association)
Amsterdam B.V. Published in The Netherlands
by Harwood Academic Publishers GmbH
Printed in Malaysia

### Surgicel® Reinforced Resection Lines in Left-sided Hepatectomy with Linear Stapling Device. An Experimental Study on Pigs

THOMAS ZILLING and TORSTEN HOLMIN

Department of Surgery, Lund University Hospital, Sweden

*(Received August 2, 1994)*

Fourteen pigs underwent left-sided hepatectomy. The resection was performed with a linear stapling device and the pigs were randomised to either Surgicel® reinforced resection lines or not.

The median time required for resection was 25 min (range 17–30) in the Surgicel® reinforced group compared to 30 min (range 21–41) in the stapled group. This difference was, however, not statistically significant ($p = 0.053$).

The postoperative haemoglobin value was lower in the stapled group compared to the Surgicel® reinforced group 69 g/l (range 42–85) versus 82 g/l (range 78–90) ($p = 0.018$). The estimated blood losses by weighing the compresses were 287 ml (range 166–379) for the stapled group and 204 ml (range 152–264) for the Surgicel® reinforced group ($p = 0.053$).

The median number of additional haemostatic sutures in the Surgicel® reinforced group was 7 (range 3–11) and in the stapled group 10 (range 5–15) ($p = 0.038$).

The haemoglobin value was similar in the two groups 1 week postoperatively; 100 g/l (range 87–104) and 102 g/l (range 95–114), $p = 0.27$, in the stapled group and the Surgicel® reinforced group, respectively. In the stapled group reinforced with the Surgicel® there was one postoperative death. In the solely stapled group there was no postoperative death ($p = 0.5$). Four out of six pigs in the Surgicel® group had massive adhesions to the resection lines. One of these six pigs was sacrificed postoperatively as it was ill and had small bowel obstruction secondary to Surgicel® induced adhesions. On the other hand, no adhesions were seen in the solely stapled pigs ($p = 0.09$). At this point, we can not recommend the use of Surgicel® to reinforce resection lines at stapled liver resection in the clinical situation, because of the high frequency of adhesions this material creates.

KEY WORDS: Liver resection stapling devices oxidised cellulose surgicel®.

### INTRODUCTION

The major problem with liver resection is control of the bleeding. This factor together with infection and liver failure are the major causes of postoperative morbidity and mortality after liver resection[1–3]. Throughout the years several techniques have been designed to limit intraoperative bleeding, such as Pringle's temporary occlusion of the portal triad[4], ultrasonic dissection[5], water jet drug dissection[6] and resection with the aid of the Nd-YAG laser[7,8].

We have previously shown that liver resection in pigs can be performed quickly and safely with a linear stapling device[9,10]. The technique has also been feasible in humans providing that the liver segment has not been too thick[10].

Regenerated oxidised cellulose (= Surgicel®) is a haemostatic web which has been used for preservation of parenchymatous organs such as the spleen[11].

Theoretically it seems attractive to combine stapling technique with Surgicel® to reinforce the resection

*Address for correspondence:* Thomas Zilling, Department of Surgery, Lund University Hospital, S-221 85 Lund, Sweden.

line and the present study compares this setting with the previous method of using a linear stapler only.

### MATERIALS AND METHODS

### Animals

Fourteen Swedish domestic pigs of random sex underwent liver resection. The median body weight in the stapled group was 20.3 kg (range 17.4–22.3) and in the Surgicel® reinforced group 20.0 kg (range 18.4–21.2). The median weight of the resected specimen was 192 g (range 167–228) in the stapled group and 171 g (range 140–199) in the Surgicel® reinforced group ($p = 0.063$). The calculated median resection size amounted to 33% (range 29–45) of the liver in the Surgicel® group and 39% (range 32–45) in the stapled group ($p = 0.43$).

### Surgical Procedure

Prior to the operative procedure the pigs were fasted for 12 hours. The pigs were anaesthetised with Pentobarbital Natrium (Abbott Scandinavia AB, Stockholm, Sweden) and endotracheal intubation was performed. The animals were artificially ventilated with a 70/30% mixture of nitrous oxide and oxygen. During the operation, one litre of Ringer-glucose (Kabi Baxter, Infusion AB, Stockholm, Sweden) was given as an infusion.

Under sterile conditions a laparotomy was performed through a midline incision. The falciform and triangular ligaments were cut. In the operating room, the pigs were randomised by closed envelopes to resection of the left part of the liver either with the aid of a RLG 90®—stapling device (Ethicon Ltd, Somerville, New Jersey, USA) or a combination of the same device with Surgicel® (Ethicon Ltd, Somerville, New Jersey, USA) reinforcement. When performing the resection with the stapler only, the most lateral segment of the left part of the liver was introduced into the jaws of the instrument. The instrument was closed and fired and the specimen cut away. A few venous or arterial bleeding points had to be ligated separately. The procedure was repeated for the resection of the medial portion of the left part of the liver. In the Surgicel® reinforced group the corresponding resection areas of the liver were covered with Surgicel®—web before the introduction into the stapler and the instrument closed and fired.

The time for performing the resection was measured, and the number of compresses weighed to give an estimation of the blood losses. Blood samples for haemoglobin was taken pre- and postoperatively.

Seven days postoperatively haemoglobin values were determined before the pigs were sacrificed and an autopsy was performed to inspect the resection area.

### STATISTICAL METHODS

Difference between groups was determined by using the Mann-Whitney U-test. The Fischer's exact probability test was used to compare frequencies.

### RESULTS

In the stapled group reinforced with the Surgicel® there was one postoperative death and one pig was killed due to being ill. In the latter pig, autopsy showed small bowel obstruction due to adhesions. Four out of six pigs in this group had massive adhesions to the resection lines. In the solely stapled group there was no postoperative death and at autopsy no adhesions were found. However, these data regarding postoperative mortality and adhesions were not statistically significant ($p = 0.5$ and 0.09, respectively).

The median time required for resection was shorter in the Surgicel® reinforced group compared to the stapled group 25 min (range 17–30) versus 30 min (range 21–41). This difference was not statistically significant ($p = 0.053$).

The median preoperative haemoglobin was 86 g/l (78–92) in the Surgicel® group and 80 g/l (72–99) in the stapled group (n.s.).

The postoperative haemoglobin value was lower in the stapled group compared to the Surgicel® reinforced group 69 g/l (range 42–85) versus 82 g/l (range 78–90), $p = 0.018$. The estimated blood losses by weighing the compresses were 287 ml (range 166–379) for the stapled group and 204 ml (range 152–264) for the Surgicel® reinforced group ($p = 0.054$).

The median number of additional haemostatic sutures in the Surgicel® reinforced group was 7 (range 3–11) and in the stapled group 10 (range 5–15) ($p = 0.038$).

There was no difference in the haemoglobin value immediately before the pigs were sacrificed after 1 week between the stapled group and the Surgicel® reinforced group (100 g/l) (range 87–104) versus 102 g/l (range 95–114), ($p = 0.27$).

### DISCUSSION

This study shows that oxidised cellulose (= Surgicel®) reinforced resection lines at stapled liver resection were beneficial in reducing operative bleeding. Although there was no significant reduction in estimated blood loss peroperatively, the postoperative haemoglobin value was significantly higher in this group compared to the solely stapled group ($p = 0.018$). This holds true also when the preoperative haemoglobin values are taken into consideration ($p = 0.38$). Furthermore, the number of additional haemostatic sutures was significantly lower in the Surgicel® group ($p = 0.038$).

Collagen based haemostatic agents have proven to be more effective than Surgicel® to prevent bleed-ing from parenchymatous organs[12–14]. However, the Surgicel®—web served as armament in our study and allowed the staples to grip into it and reinforce the resection line. We do think that this is the most natural explanation to the reduced operative blood loss in this group. We did also try native collagen from bovine tendon (Collastat®) for the same purpose. But this material only broke into pieces and could not serve as armament for the staples in the resection line.

In the Surgicel® group one pig died postoperatively of unknown reason. Four out of six pigs had massive adhesions to the resection area. One pig was sacrificed due to being ill and had small bowel obstruction due to Surgicel® related adhesions. No significance was obtained neither regarding postoperative mortality ($p = 0.5$) nor frequency of adhesions ($p = 0.09$). In the literature some authors have reported that Surgicel® is able to prevent peritoneal adhesions, whereas others have come to the reverse conclusion[15,16]. According to Pierce and co-workers Surgicel® consists of a soluble uronic acid component and a fibrous material which needs phagocytosis to be removed. This could explain the increased number of adhesions observed[17]. Another drawback for Surgicel® is the increased risk for infection observed experimentally which also can increase the risk for adhesions and postoperative morbidity[18]. Although operative bleeding is reduced by using Surgicel® at stapled liver resection in pigs, we cannot recommend Surgicel® to be used in the clinical situation, because of the high frequency of Surgicel® related adhesions obtained in the present study.

### ACKNOWLEDGEMENTS

This study was supported by grants from Johnsson & Johnsson A. B., Stockholm and the Medical Faculty, Lund University, Lund, Sweden.

### REFERENCES

1. Ekberg, H. (1986) Colorectal Liver Cancer, Resection and Regional Chemotherapy. *Thesis*, Lund, Sweden.
2. Thompson, H. H., Tompkins, R. K., Longmire, W. P. (1983) Major Hepatic Resection. A 25-year experience. *Ann. Surg.*, **197**, 375–388.
3. Iwatsuki, S., Shaw, B. W., Starzl, T. E. (1983) Experience with 150 liver resections. *Ann. Surg.*, **197**, 247–253.
4. Walt, A. J., Bender, J. S. (1985) Injuries of the liver from Maingot's Abdominal Operations, eighth edition, pp. 1577–1590, ISBN 0-8385-6099-7.
5. Hodgson, W. J., DelGuecio, L. R. (1984) Preliminary Experience in Liver Survery by Using the Ultrasonic Scalpel. *Surgery*, **95**, 230–234.
6. Persson, B. G., Jeppsson, B., Tranberg, K. G., Roslund, K., Bengmark, S. (1989) Transection of the Liver With a Water Jet. *Surg. Gynecol. Obstet.*, **168**, 267–268.
7. Joffe, S. N., Brackett, K. A., Sankar, M. Y., Daikuzono, N. (1986) Resection of the Liver with the Nd: YAG laser. *Surg. Gynecol. Obstet.*, **163**, 437–442.
8. Tranberg, K.G., Rigotti, P., Brackett, K.A., Björnson, H. S., Fischer, J. E., Joffe, S. N. (1986) Liver Resection. A Comparison Using the Nd-YAG laser, an Ultrasonic Surgical Aspirator, or Blunt Dissection. *Am J. Surg.*, **151**, 368–373.
9. Zilling, T., Walther, B. S., Holmin, T. (1990) Segmental Liver Resection With Linear Stapling Devices. In Vivo, **4**, 273–276.
10. Zilling, T., Walther, B.S., Holmin, T. (1992) Left-Sided Hepatectomy with a Linear Stapling Device. An Experimental Study on Pigs. *HPB Surgery*, **6**, 51–55.
11. Aidonopoulus, A., Papavramidis, S., Goutzamanis, G., Filos, G., Deligiannidis, N., Hanos, G. (1993) A simple and safe method for preservation of the injured spleen. *Injury*, **24**, 300–302.
12. Zoucas, E., Göransson, G., Bengmark, S. (1984) Comparative evaluation of local hemostatic agents in experimental liver trauma: a study in rat. *J. Surg. Res.*, **37**, 145–150.
13. Raccuia, J., Simonian, G., Dardik, M., Hallac, D., Raccuia, S., Stahl, R., Dardik, H. (1992) Comparative efficacy of topical hemostatic agents in a rat kidney model. *Am. J. Surg.*, **163**, 234–238.
14. Blair, S., Backhouse, C., Harper, R., Matthews, J., McCollum, C. (1988) Comparison of absorbable materials for surgical haemostatis. *Br. J. Surg.*, **75**, 969–971.
15. Larsson, B., Nisell, H., Granberg, I. (1978) Surgicel®—An absorbable hemostatic material—in prevention of peritoneal adhesions in rats. *Acta Chir. Scand.*, **144**, 375–378.
16. Yemini, M., Meshorer, A., Katz, Z., Rozenman, D., Lancet, M. (1984) Prevention of reformation of pelvic adhesions by "barrier" methods. *Int. J. Fertil.*, **29**, 194–196.
17. Pierce, A., Wiebkin, O., Wilson, D. (1984) Surgicel its fate following implantation. *J. Oral Pathol.*, **13**, 661–670.
18. Scher, K., Coil, J. (1982) Effect of oxidized cellulose and microfibrillar collagen on infection. *Surgery*, **91**, 301–304.

